# Dynamic changes in protein interaction between AKAP95 and Cx43 during cell cycle progression of A549 cells

**DOI:** 10.1038/srep21224

**Published:** 2016-02-16

**Authors:** Xiaoxuan Chen, Xiangyu Kong, Wenxin Zhuang, Bogang Teng, Xiuyi Yu, Suhang Hua, Su Wang, Fengchao Liang, Dan Ma, Suhui Zhang, Xuan Zou, Yue Dai, Wei Yang, Yongxing Zhang

**Affiliations:** 1 State Key Laboratory of Molecular Vaccinology and Molecular Diagnostics, School of Public Health, Xiamen University, Xiamen, Fujian, 361102, PR China; 2 Affiliated Zhongshan Hospital of Dalian University, Dalian, Liaoning, 116001, PR China; 3 The First Affiliated Hospital of Xiamen University, Xiamen, Fujian, 361102, PR China; 4 School of Life Science and Bio-pharmaceutics, Shenyang Pharmaceutical University, Shenyang liaoning, 110016, PR China

## Abstract

Here we show that A-kinase anchoring protein 95 (AKAP95) and connexin 43 (Cx43) dynamically interact during cell cycle progression of lung cancer A549 cells. Interaction between AKAP95 and Cx43 at different cell cycle phases was examined by tandem mass spectrometry(MS/MS), confocal immunofluorescence microscopy, Western blot, and co-immunoprecipitation(Co-IP). Over the course of a complete cell cycle, interaction between AKAP95 and Cx43 occurred in two stages: binding stage from late G1 to metaphase, and separating stage from anaphase to late G1. The binding stage was further subdivided into complex binding to DNA in interphase and complex separating from DNA in metaphase. In late G1, Cx43 translocated to the nucleus via AKAP95; in anaphase, Cx43 separated from AKAP95 and aggregated between two daughter nuclei. In telophase, Cx43 aggregated at the membrane of the cleavage furrow. After mitosis, Cx43 was absent from the furrow membrane and was located in the cytoplasm. Binding between AKAP95 and Cx43 was reduced by N-(2-[P-Bromocinnamylamino]-ethyl)-5-isoquinolinesulfonmide (H89) treatment and enhanced by Forskolin. dynamic interaction between AKAP95 and Cx43 varies with cell cycle progression to regulate multiple biological processes.

AKAP95 is a member of the AKAP family of proteins, which are mainly located in the nucleus of mammalian cells. In interphase, AKAP95 is primarily bound to the nuclear matrix. Some AKAP95 is bound to chromatin, but none is present in the nucleolus. At the beginning of mitosis, the majority of AKAP95 is relocated to chromatin^1^. The processes of chromatin targeting and binding, as well as chromosome condensation, are controlled by the activity of AKAP95 in a zinc finger-dependent manner^2^. The recruitment of hCAP-D2/Eg7 to chromosomes by AKAP95 also contributes to the above processes^3^, during which protein kinase A (PKA) is not involved^4^.

AKAP95 functions as a scaffold to integrate protein signaling complexes to cellular outputs^5^. For example, AKAP95 has been shown to form complexes with p68 RNA helicase, RSK1, and MCM2 in the nuclear matrix; these interactions help regulate DNA replication^5^,^6^,^7^ and maintain mRNA stability^8^ in the rat brain. In addition, AKAP95 regulates mitosis^9^ and apoptosis^10^ via histone modifications. AKAP95 also regulates gene expression via MLL2-mediated histidine H3K4 methylation^11^.

AKAP95 can influence cell cycle progression by binding to cyclins D_1-3_/E_1_^4^,^12^. G1/S cyclins interact with the RII subunit of PKA through AKAP95. Interestingly, the binding of cyclins to AKAP95 can be substituted by CDKs; for example, CDK4 instead of cyclin D_3_ and CDK2 for cyclin E_1_^4^,^12^.

Connexin 43 (Cx43) belongs to the family of connexins, which regulate cell growth and proliferation via gap junction intercellular communication. The C terminus of Cx43 contains multiple regulatory phosphorylation sites. Cx43 has been shown to interact with multiple proteins, including cadherins, occludin, ZO-1, ZO-2, α-/β-catenins, and CIP75. Through several of these interactions, the phosphorylation state of Cx43 is altered, which, in turn, regulates the function of gap junction channels^13^,^14^,^15^,^16^,^17^,^18^,^19^. Overexpression of Cx43 inhibits G1 to S phase progression^20^,^21^,^22^,^23^,^24^, extends the duration of mitosis, and blocks G1 phase^25^. In addition, Cx43 reduces Skp2 expression, inhibits CDK2- and CDK4-mediated phosphorylation of Rb, and regulates cell proliferation by binding cyclin E^26^.

While Cx43 is a tumor suppressor, AKAP95 promotes tumor growth^27^. Such processes are tightly linked to cell cycle control via the activity of cyclin-CDK complexes^4^,^12^,^20^,^26^. Previously, we demonstrated a correlation between expression of AKAP95 and Cx43 in lung cancer^27^,^28^. Those findings suggested that these two proteins might interact and affect cell cycle progression by regulating the activity of cyclins and CDKs. In the present study, we provide evidence that the interaction between AKAP95 and Cx43 is dynamically regulated in lung cancer cells during cell cycle progression.

## Materials and Methods

### Reagents and Materials

Mouse anti-AKAP95 (22-Z, SC-100643) monoclonal antibody, mouse anti-Cx43 (D-7, SC-13558) monoclonal antibody, rabbit anti-Cx43 (H-150,SC-9059) polyclonal primary antibody, GADPH (SC-110976) primary antibody, and protein A/G Plus-Agarose beads (SC-2003) were obtained from Santa Cruz (Dallas, Texas, USA). Mouse anti-β-tubulin (1879-1) primary antibody was purchased from Epitomics (Burlingame, CA, USA). LaminB1 (BS3547) primary antibody was obtained from BioWorld (Nanjing, Jiangshu, China). GADPH (AB90090) primary antibody was obtained from Sangon Biotech Co., Ltd (Shanghai, China). L-mimosine (0253), aphidicolin (A0781), nocodazole (M1404), colchicine (C9754), H89 dihydrochloride hydrate (B1427), Forskolin (F6886), and Dimethyl sulfoxide (DMSO) were purchased from Sigma (Santa Clara, CA, USA). Alkaline phosphatase (#EF0651) was purchased from Thermo Scientific (Waltham, MA, USA). DMEM/High Glucose (SH30243.01B) and fetal bovine serum (SH300 84.03HI) were purchased from HyClone (Logan, UT, USA). Cell lysis buffer for western blot and IP was purchased from Beyotime Institute of Biotechnology (Haimen, Jiangsu, China, P0013). Nuclear-cytosol extraction kit was purchased from Applygen Technologies Inc (Beijing, China, P1200). Proximity ligation assay technology kit was purchased from Sigma (Santa Clara, CA, USA, DU092101).

Cx43 gene was cloned into the pcDNA3.1 ( + ) vector. And the pcDNA3.1 ( + )-Cx43 plasmid was transfected into A549 cell line by lipofectamine2000. The stable Cx43-expressing cells were selected by G418.

### Mass spectrometry(MS/MS) *assay*

ESI-MS(LTQ) analysis was performed at the Shanghai Institute for Biological Sciences, Chinese Academy of Science, Shanghai, China.

### Cell culture

A549 and L-O2 cells were cultured in Dulbecco’s modified Eagle’s medium (DMEM) with 5% CO_2_ and 90% humidity at 37 °C.

### Immunofluorescehnce microscopy

When cells were 60% confluent, DMEM medium was discarded. Coverslips for cell culture were washed three times with phosphate-buffered saline, fixed in pre-cooled ethanol (4 °C) for 15 min, and washed again with PBS. The coverslips were treated with 0.5% TritonX-100 at room temperature for 20 min and blocked with 1% BSA at 37 °C for 1 h. The specimens were then incubated with primary antibodies (1:100) at 4 °C overnight followed by incubation with FITC- and TRITC-labeled secondary antibodies (1:100) in the dark at 37 °C for 2 h. The nucleus was stained with 0.5 μg/mL DAPI. Cell fluorescence imaging was examined using a Zeiss LSM5 confocal laser scanning microscope.

### Proximity ligation assay

Consecutive cell slices were incubated in 0.5% TritonX-100 at RT for 15 min and then blocked with 1% BSA for 60 min^29^,^30^. Antibodies directed against Cx43 and AKAP8 were incubated overnight at 4 °C. PLA probes were then added for 60 min at 37 ^o^ C. The slices were incubated with a mixed solution of 1:40 linking ligase agent diluted in linking agent. The linking agent was then diluted 1:5 with ultrapure water at 37 ^o^ C for 30 min. Next, the polymerase was diluted 1:80 with amplifying solution, and, finally, the amplifying solution was diluted 1:5 with ultrapure water. FITC-labeled anti-laminB1 antibody was used for marking the nuclear membrane. Finally, the slices were incubated with Duolink *In Situ* reagent containing DAPI and detected by laser scanning confocal microscope (LSM5, Zeiss).

### Usage of blockers

L-mimosine^31^,^32^ and colchicine^33^,^34^ were prepared in DMEM. Aphidicolin^35^ and nocodazole were prepared in DMSO. Gradient concentrations of the blockers were added to A549 and L-O2 cell cultures. After 24 h, cell cycle phases were examined by flow cytometry to select the optimal dose as the working concentration.

### Use of H89 and Forskolin

Solutions were prepared in DMSO at a concentration of 20 μM^36^,^37^,^38^,^39^.

### Alkaline phosphatase(ALP) treatment

Total protein was diluted with radio immunoprecipitation assay buffer to a final concentration of 0.2 μg/μL. Samples were divided into two groups (500 μL each). Following the manufacturer’s instructions, 50 μL of FastAP buffer with 250 U of ALP was added into one group, and 50 μL of FastAP buffer with 250 μL of ultrapure water was added into the other group. The two treated groups of cells were then incubated at 37 °C for 1 h before performing Co-IP and Western blot assays.

### Separation of nuclear and cytoplasmic proteins

Nuclear and cytoplasmic proteins were extracted according to the instructions provided with the Nuclear-Cytosol Extraction Kit. Briefly, cells were collected, added with the CEB-A solution, mixed and vortexed, and then added with the CEB-B solution, mixed and vortexed again, followed by centrifugation. The supernatant contained the cytoplasmic proteins (including several nuclear membrane proteins, if CEB-B were not added, there would be no nuclear membrane protein) and the precipitant was further added with the NEB solution, incubated, vortexed and centrifuged. The resultant supernatant contained nuclear proteins.

### Western blot

Once cells were 80% confluent, they were lysed with appropriate buffer. Protein concentrations were determined using the Pierce BCA Protein Assay kit (NCI1059CH, Thermo, Waltham, MA, USA) and then adjusted to the same level. Proteins were denatured by heating at 100 °C for 5 min, subjected to SDS-PAGE electrophoresis, and transferred to membranes. For immunoblotting, the membranes were incubated with primary antibodies at 4 °C overnight and then with secondary antibodies for 1 h at room temperature. Enhanced chemiluminescent substrate was added for gel imaging or film exposure. Images were collected using a Chemi DOC XRS + Imaging System (BioRad, Hercules, CA, USA). The gray scale value of protein bands was scanned using Image Lab5.0 (Biorad, Hercules, CA, USA).

### Co-immunoprecipitation asssay

Three micrograms of primary antibody was added to 500 μg of extracted proteins. The mixture was incubated at 4 °C for 60 min with gentle mixing. Then, 20 μl of Protein A/G Plus-Agarose beads was added and incubated at 4 °C overnight. The mixture was centrifuged at 2,500 rpm for 5 min at 4 °C. The supernatant was discarded, and the Co-IP products were washed three times with PBS. After the final wash, the precipitates were re-suspended in 40 μL of sample buffer for western blot assay.

### Statistical analysis

The gray value of protein bands was analyzed by Kruskal-Wallis H of Nonparametric test in SPSS21.0 (SPSS Inc., Chicago, IL, USA,20131125-ZSXM).

## Results

### Identification of Cx43 binding partners in lung cancer cells detected by MS/MS assay

**Figure 1:**
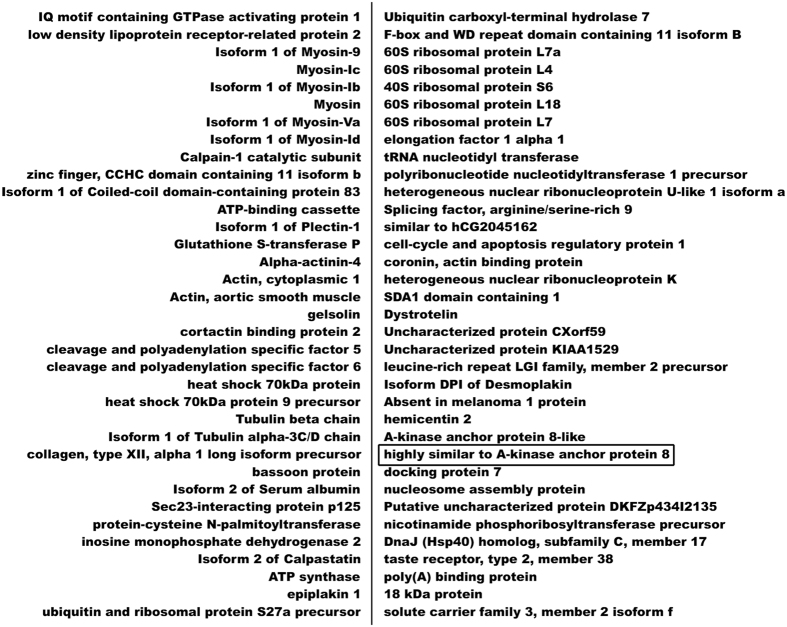
Proteins interacting with Cx43 in A549 cells detected by MS/MS analysis.

Co-IP experiments were carried out in A549 cells using an anti-Cx43 antibody. Co-IP products were analyzed by LTQ tandem mass spectrometry. In total, 72 proteins involved in cell structural integrity, cell motility, cytokinesis, cell cycle, and apoptosis were identified as Cx43-interacting partners (Fig. 1). The proteins were classified and analyzed in terms of biological function, and AKAP95 was selected as a promising target for additional analysis.

### Binding between AKAP95 and Cx43 in lung cancer cells detected by cytological assays

**Figure 2:**
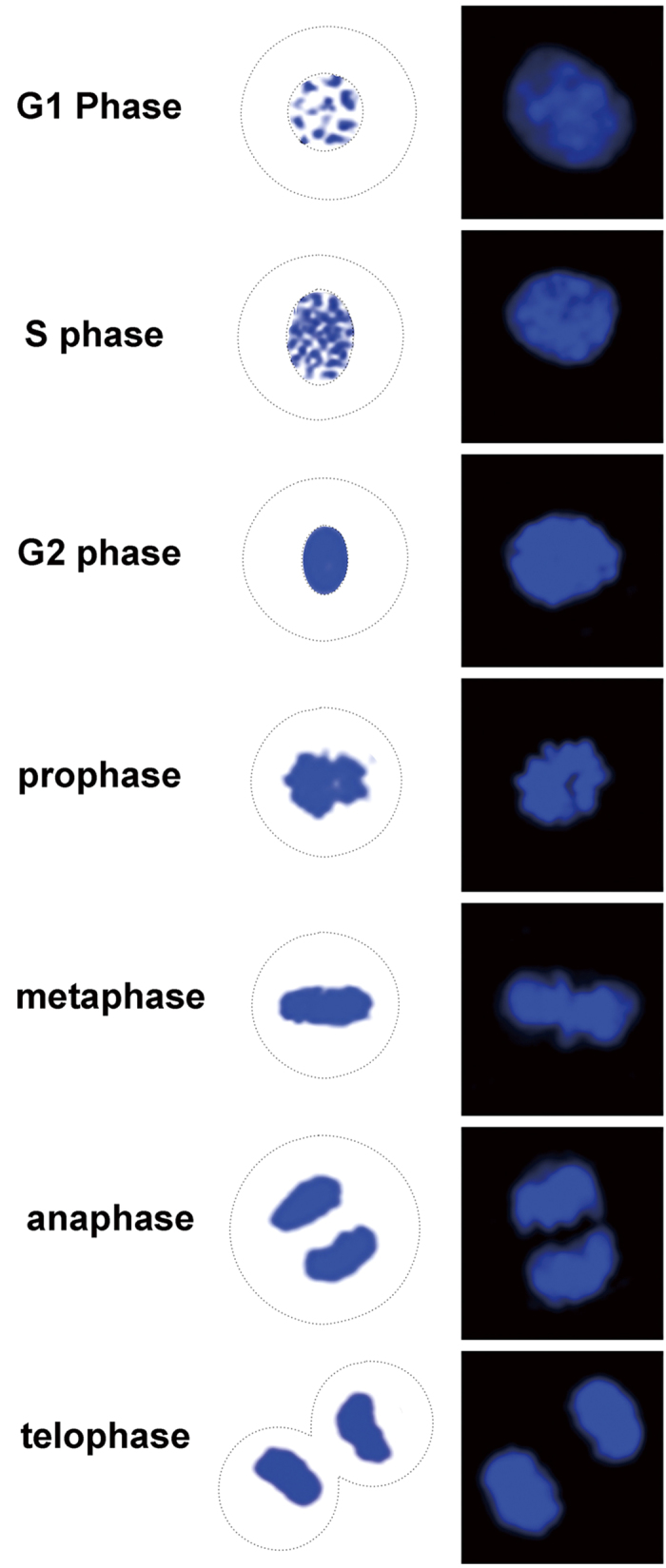
Nuclear morphology of different cell cycle phases.

**Figure 3:**
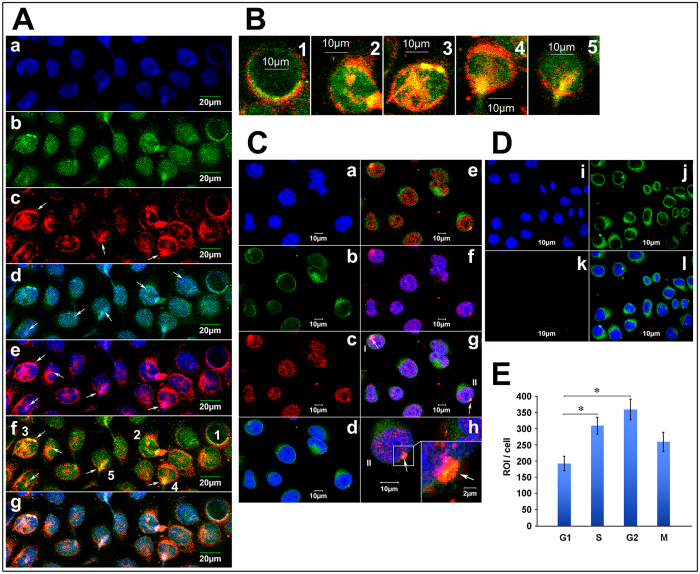
Co-localization of Cx43, AKAP95 and DNA in A549 cells (**A**) Co-localization of Cx43 and AKAP95 in the nucleus. (Aa) Blue fluorescence represents DAPI-labeled nuclei; (Ab) green fluorescence represents FITC-labeled AKAP95; (Ac) red fluorescence represents TXRD-labeled Cx43; (Ad) merged image of Aa and Ab; (Ae) merged image of Aa and Ac; (Af) merged image of Ab and Ac; and (Ag) merged image of Aa, Ab, and Ac; (**B**) Rows 1–5 are the enlarged images of cells 1–5 in Af. (magnification 200 × for A; 200 × 4 for B). (**C**) Proximity ligation assay and immunofluorescence detected by Laser scanning confocal microscope (200 × ). Labeling with DAPI is shown in (Ca), and lamin B1 is shown in (Cb). In (Cc), the combination of AKAP95 and Cx43 was labeled with TXRD. Results showed interaction between AKAP95 and Cx43 (red). Both proteins were localized in the nucleus. Panel (Cd) is a merged image of (Ca) and (Cb). Panel (Ce) is a merged image of (Cb) and (Cc). Panel (Cf) is a merged image of (Ca) and (Cc). In panel (Cg) (merged image of Ca, Cb and Cc), cells highlighted as I and II point to the combination of AKAP95 and Cx43 crossing the nuclear membrane. Cells II are enlarged in (Ch) (200 × 4). At the lower right corner (200 × 4 × 4), the junction between complex and membrane was demonstrated by yellow fluorescence(arrow), suggesting movement into the nucleus via the nuclear membrane. (**D**) is the negative control to C. (Di) is DAPI, (Dj) is Lamin B1, (Dk) is Cx43 primary antibody replace by PBS as a negative control in PLA assays; red fluorescence was not detected. Panel (Dl) is a merged image of (Di), (Dj) and (Dk). (**E**) We detected the quantitative changes of AKAP95/Cx43 complexes in G1, S, G2, M phases using Olympus software in PLA assays. The number of the detected cells was 37 in G1 phase, 30 in S phase, 13 in G2 phase and 10 in M phase. In addition, there were statistically significant differences of the amount of AKAP95/Cx43 complexes between G1 and S phase, G1 and G2 phase respectively.

Cell cycle phases were determined by nuclear morphology (Fig. 2) and staining intensity of AKAP95 and Cx43 proteins. The levels of AKAP95 and Cx43 in A549 cells were detected by fluorescent immunocytochemistry and confocal laser scanning microscopy (Fig. 3).

Cells labeled with blue-fluorescent DAPI were considered to be in late G1 or early S phase (G1/S) (Fig. 3Aa). AKAP95 (labeled with green-fluorescent FITC) was mainly expressed in the nucleus (Fig. 3Ab). Cx43 (labeled with red-fluorescent TXRD) was mainly expressed in the cytoplasm, Cx43 was expressed in the nucleus in the form of bright and coarse speckles (Fig. 3Ac, arrows). The patchy, pale-green fluorescence represents AKAP95 bound to DNA (Fig. 3Ad, arrows point to), and the patchy pink fluorescence represents Cx43 bound to DNA (Fig. 3Ae, arrows). Yellow fluorescence was observed in the nucleus (Fig. 3Af, corresponding to patchy pink fluorescence in Fig. 3Ae) but not in the cytoplasm, suggesting that Cx43 and AKAP95 co-localize in the nucleus only. Taken together, the above observations indicate co-localization of AKAP95, Cx43, and DNA within the nucleus.

The image of the cells (labeled 1–5) in Fig. 3Af is further enlarged to higher resolution (Fig. 3B, columns 1–5). The inner and outer nuclear membranes are stained green and red, respectively, and yellow fluorescence is shown in the nucleus and nuclear membrane (Fig. 3B, columns 1–5, arrows).

AKAP95 contains a bipartite nuclear localization signal and a nuclear matrix targeting sequence; in contrast, Cx43 has no nuclear recognition sequence. Thus, their co-localization in the nuclear membrane suggests that AKAP95 may function as a carrier protein of Cx43 for its transport to the nucleus. Three cells (Fig. 3B, 3–5), may demonstrate the typical process of AKAP95 and Cx43 binding and import into the nucleus. The proteins first bound to the nuclear membrane and highly aggregated into patches (Fig. 3B3); the protein patches then started to diffuse into the nucleus (Fig. 3B4) and the proteins were finally imported into the nucleus (Fig. 3B5).

The proximity ligation assay was performed to further verify the formation of AKAP95/Cx43 complexes in the nuclear membrane and entry into the nucleus (Fig. 3C). The nuclear membrane is labeled with FITC-conjugated anti-lamin B1 (Fig. 3Cb). Results from the proximity ligation assay showed that AKAP95 and Cx43 were both located in the nucleus in a punctate pattern shown in red (Fig. 3Cc). No red fluorescence was detected for the corresponding negative control (Dk). There was no detection of these proteins in the cytoplasm (Fig. 3Ce). Pink fluorescence indicated co-location of AKAP95, Cx43 and DNA in the nucleus (Fig. 3Cf). AKAP95/Cx43 complexes formed in the nuclear membrane, and were capable of crossing the nuclear membrane (Fig. 3Ce,g,h (arrows); 3Ch shows an enlarged image of cell II shown in 3Cg). The junction between the complexes (red fluorescence) and the nuclear membrane (green fluorescence) showed yellow fluorescence (Fig. 3Ch, lower right corner, arrows), suggesting that AKAP95, after binding Cx43, passed into the nucleus through the nuclear membrane. Additionally, we observed that cells with yellow fluorescence in the nuclear membrane were all in G1/S phase and not in G2 phase, indicating that AKAP95 may transport Cx43 to the nucleus specifically within the brief period of G1/S phase. The results of the PLA detection showed that AKAP95/Cx43 complexes in G1 phase were the least. The complexes gradually increased in S phase and G2 phase, while decreased in M phase (Fig. 3E).

**Figure 4:**
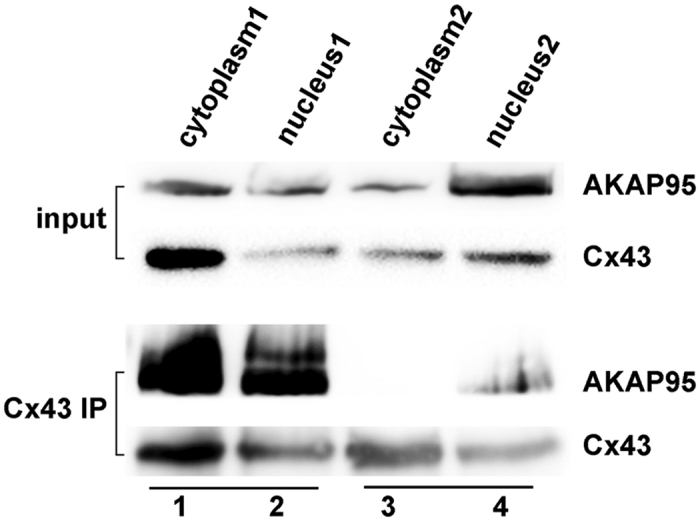
Co-localization of AKAP95 and Cx43 in the nuclear membrane. Using nuclear-cytosol extraction kits according to the manufacturer’s instructions, cytoplasmic and nuclear fractions were separated by using two of protocols: 1. Adding CEB-B solution to produce the Nucleus1 and Cytoplasm1 fractions containing nuclear membrane proteins; 2. Without adding CEB-B solution to produce the Nucleus2 and Cytoplasm2 fractions without nuclear membrane proteins. Samples (300 μg) of each group were subjected to Co-IP using an anti-Cx43 antibody (3 μg). Immunoprecipitated lysates (100 μg) were then analyzed by Western blotting using anti-AKAP95 and anti-Cx43 antibodies. The experiment was repeated three times.

Cytoplasmic and nuclear fractions from A549 cells were separated using Nuclear-cytosol extraction kit. Two protocols were used for the separation: 1. Adding CEB-B solution to produce the Nucleus1 and Cytoplasm1 fractions containing nuclear membrane proteins; 2. Without adding CEB-B solution to produce the Nucleus2 and Cytoplasm2 fraction without nuclear membrane proteins. The extracted proteins were subjected to Co-IP using an anti-Cx43 antibody. Immunoprecipitated lysates were then analyzed by Western blotting using anti-AKAP95 and anti-Cx43 antibodies. The interaction between AKAP95 and Cx43 protein was detected in Cytoplasm1 containing proteins of nuclear membrane. In contrast, although the Cx43 protein and a small quantity of AKAP95 protein were present in the cytoplasm, their interaction was not detected in Cytoplasm2 which did not contained nuclear membrane proteins (Fig. 4, lanes 1 and 3). Their interaction was detected in nuclear fractions with or without nuclear membrane proteins (Fig. 4, lanes 2 and 4). These results indicate that Cx43 binds to AKAP95 in the nucleus and nuclear membrane.

Together, the above results demonstrate that: 1) AKAP95 might be the transporter that carries Cx43 to the nucleus in A549 cells during G1/S phase; 2) In the nucleus, Cx43 simultaneously binds both AKAP95 and DNA; their co-localization implies that the Cx43-AKAP95 protein complex might directly bind to DNA and further regulate DNA expression or participate in DNA aggregation and condensation; and 3) The amount of AKAP95/Cx43 complexes increased along with the progress of G1, S and G2 phase, while decreased in M phase.

### Dynamic interaction between AKAP95 and Cx43 during the cell cycle

**Figure 5:**
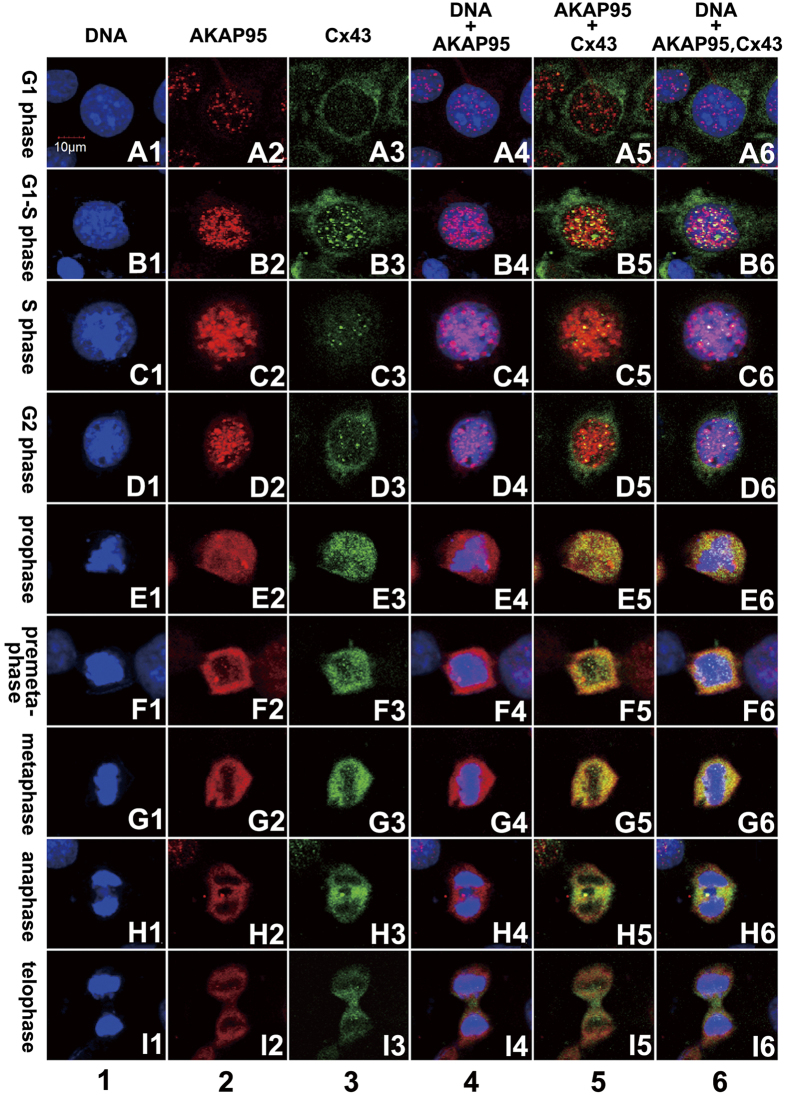
Expression, localization, and co-localization of AKAP95 and Cx43 in A549 cells during different cell cycle phases. Column 1 (A1-I1): DAPI-labeled nuclei with morphological changes during a complete cell cycle from G1 to telophase; column 2 (A2-I2): expression and localization of TRITC-labeled AKAP95 in each cell cycle phase; column 3 (A3-I3): expression and localization of FITC-labeled Cx43 in each cell cycle phase; column 4 (A4-I4): merged images of columns 1 and 2; column 5: merged images of columns 2 and 3; and column 6: merged images of columns 1, 2, and 3 (200 × 3). The experiment was repeated four times.

Co-localization of AKAP95 and Cx43 at different cell cycle phases was examined in both A549 cells and human gastric adenocarcinoma BGC823 cells by double-labeling immunofluorescence (i.e., AKAP95 labeled with TRITC, and Cx43 labeled with FITC). Samples were examined by confocal laser scanning microscopy, and cell cycle phases were determined according to nuclear morphology (Fig. 2) combined with the expression of AKAP95 and Cx43 protein. The total count of A549 cells at different stages was 566, including 495 in G1/S phase, 17 in G2 phase, 24 in prophase, 15 in metaphase, 9 in anaphase, and 6 in telophase. The results from A549 cells are presented in Fig. 5.

In A549 cells, AKAP95 and Cx43 were expressed at low levels in G1 phase. Protein expression gradually increased in G1-S phase and even more so in S-metaphase. The highest expression of AKAP95 and Cx43 was observed in G2-metaphase. Protein expression, particularly that of AKAP95, was decreased beginning from anaphase, and decreased to the lowest level in telophase (Fig. 5, columns 2 and 3). AKAP95 was mainly expressed in the nucleus (Fig. 5, column 2), and Cx43 was mainly expressed in the cytoplasm (Fig. 5, column 3). However, low levels of Cx43 could be detected in the nucleus in G1 phase; this increased in G1/S phase (Fig. 5, column 3). These results supported that Cx43 is transported to the nucleus by AKAP95 during a short period in G1/S phase.

AKAP95 binds to chromatin in G1-G2 phase; during mitosis, this protein mostly separates from chromatin but remains in the vicinity of the chromosomes. AKAP95 substantially aggregates between two daughter nuclei in anaphase; a few spots of pink fluorescence appeared in the chromosome during mitosis, suggesting that a small amount of AKAP95 was bound to DNA during this stage (Fig. 5, column 4). Yellow fluorescence was not observed in the nucleus in G1 phase but appeared during the G1/S phase to anaphase transition (Fig. 5, column 5). This indicates that AKAP95 is constantly bound to Cx43 during G1/S phase to anaphase; their binding capacity began to gradually increase in G1/S, peaked in prophase and pre-metaphase, and then decreased afterward, finally separating in telophase.

Our data indicate that Cx43, AKAP95 and DNA are bound together in G1/S-G2 phase. This interaction is gradually lost in mitosis, as the two proteins gradually separate from DNA but remained bound to one another and surrounding DNA. The binding capacity of Cx43 and AKAP95, which remain bound to one another and surrounding DNA, begins to decrease in anaphase, and these two proteins relocate to the space between two daughter nuclei. Additionally, expression of Cx43 is higher than that of AKAP95. No complex was detected in telophase, and Cx43 was translocated from the middle of two daughter nuclei to the membrane site of the cleavage furrow. Similar results were observed in the gastric cancer cell line BGC-823 (data not shown).

**Figure 6:**
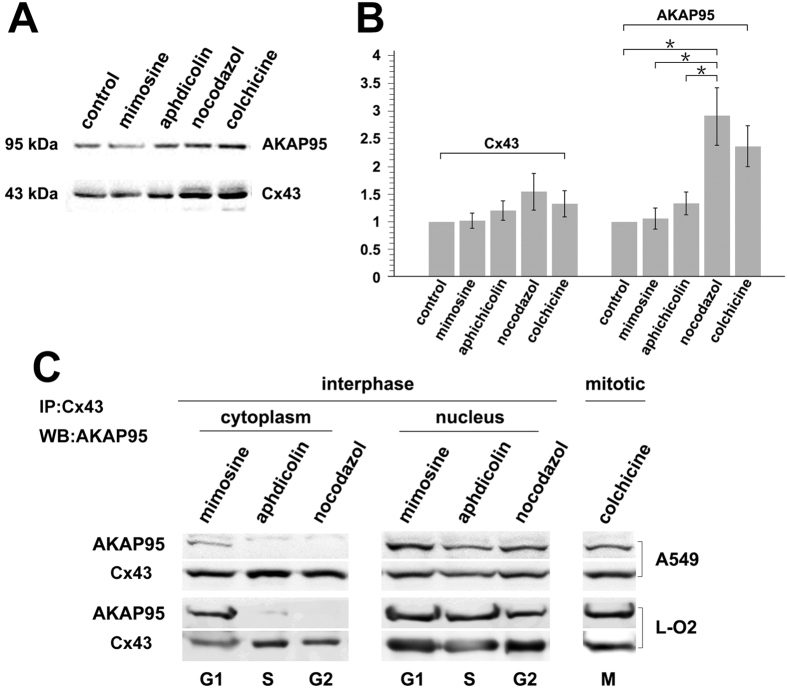
Interaction between Cx43 and AKAP95 in A549 and L-O2 cells at different cell cycle phases. A549 and L-O2 cells were treated with L-mimosine (G1) (75 and 80 μg/mL), aphidicolin (S) (0.8 and 1 μg/mL), nocodazole (G2) (0.6 and 1 μg/mL), and colchicine (M) (0.5 and 0.5 μg/mL) for 24 h. A portion of the cells were subjected to total protein extraction and analysis. (**A**) Changes in AKAP95 and Cx43 expression determined by Western blot; and (**B**) statistical analysis of AKAP95 and Cx43 expression levels derived from images acquired by the BioRad Chemi DOC XRS + Imaging System. The gray value of protein bands was scanned using Image Lab5.0, and the ratio of band intensity in the treatment group versus the control group was determined using one-way analysis of variance in SPSS 21.0. * indicates statistically significant difference between groups at *p* < 0.05. Remaining cells were subjected to cell fractionation. (**C**) Co-immunoprecipitation assay using anti-Cx43 antibody and Western blot using anti-AKAP95 antibody, when cytoplasm and nuclear protein were separated, a small amount nuclear membarnce protein was ramained in the cytoplasm protein. The experiment was repeated four times.

A549 cells and the fetal hepatocyte cell line L-O2 were treated with the following inhibitors for 24 hr: L-mimosine (G1), aphidicolin (S), nocodazole (G2), and colchicine (M). AKAP95 and Cx43 protein expression in each of the treatment groups was examined by Western blot analysis (Fig. 6A,B). Additionally, nuclear and cytoplasmic fractions were obtained from treated cells, and Co-IP was performed using an anti-Cx43 antibody followed by Western blot detection of AKAP95 (Fig. 6C).

AKAP95 and Cx43 expression were gradually increased from G1, S, to G2 phase; their expression was decreased during mitosis (Fig. 6A). The lowest protein expression levels were observed in G1 phase. The highest levels were observed in G2. AKAP95 expression fluctuated more dramatically than Cx43. Specifically, AKAP95 was 2.8-fold higher in G2 phase compared to control (Fig. 6B; *p* < 0.05).

Interaction between AKAP95 and Cx43 was observed in the cytoplasm of A549 and L-O2 cells only in G1 phase (Fig. 6C). Because nuclear membrane proteins were released into the cytoplasm when we separated cytoplasmic and nuclear proteins, we could only make observations in G1 phase of the cell cycle. These findings are consistent with the results of confocal immunofluorescence (Fig. 3). Thus, Cx43 is likely shuttled to the nucleus by AKAP95 in late G1 phase. In the nucleus, AKAP95 and Cx43 were found bound together in G1, S, G2, and M phases, which is also consistent with our earlier data (Fig. 5, column 5, B5-G5).

Our data indicate that the binding between AKAP95 and Cx43 is dynamic and tightly cell cycle regulated.

### Binding between AKAP95 and Cx43 is affected by PKA activity

To explore whether PKA phosphorylation affected the binding between AKAP95 and Cx43, H89 and Forskolin were used to inhibit and activate PKA activity, respectively. A549 cells were treated with either drug for 24 h, and the levels of AKAP95 and Cx43 were detected by Western blot.

**Figure 7:**
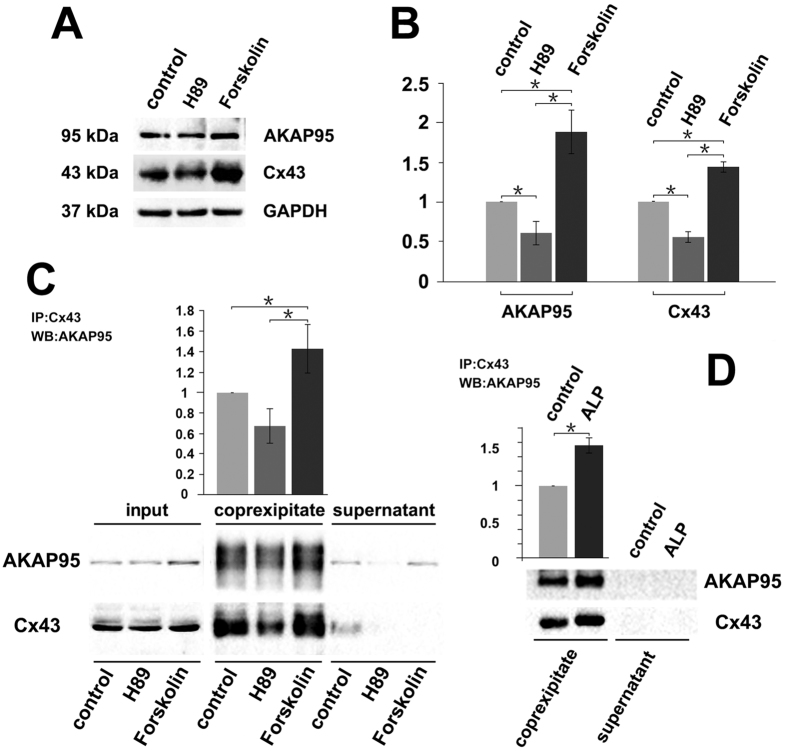
Binding between AKAP95 and Cx43 is affected by PKA activity. (**A**) Western blot examination of Cx43 and AKAP95 expression; GAPDH used as the internal reference. Each lane contains 30 μg of total proteins. The experiment was repeated three times. (**B**) Statistical data of AKAP95 and Cx43 expression levels in cells after H89 and Forskolin treatments (20 μM each). AKAP95 and Cx43 expression levels decreased in the H89 treatment (*p* < 0.05) and increased in the Forskolin treatment compared to the control group (*p* < 0.05). Images were acquired using the BioRad Chemi DOC XRS + Imaging System, and the gray value of protein bands was scanned using Image Lab5.0. The ratio of measurements of the treatment group to the control group was calculated by one-way analysis of variance in SPSS 21.0. (**C**) Western blot analysis of binding between AKAP95 and Cx43 in cells after H89 and Forskolin treatments. 500 μg of extracted total proteins was co-immunoprecipitated (Co-IP) with 3 μg of anti-Cx43 antibody. The Co-IP products were resuspended in an equal volume of loading buffer, and the supernatant was subjected to western blot with anti-Cx43 and anti-AKAP95 antibodies, and the grayscale value of the bands of the H89 and forskolin groups were compared with those of the control bands and the ratios were subjected to statistical analysis. (**D**) Western blot analysis of binding between AKAP95 and Cx43 after alkaline phosphatase (ALP) treatment. 500 μg of extracted total proteins was diluted to 0.2 μg/μL with RIPA lysis buffer and split into two groups (500 μL each). The ALP treatment was performed following the manufacturer’s instructions (see *ALP treatment* in M&M). The protein diluents were subjected to Co-IP with 1 μg of anti-Cx43 antibody, and the products were resuspended in an equal volume of loading buffer, and then were subjected to western blot analyzed with anti-Cx43 and anti-AKAP95 antibodies, the grayscale value of the bands of ALP treatment groups were compared with those of the control bands and the ratios were subjected to statistical analysis. Results showed that the binding capacity of Cx43 and AKAP95 was improved after ALP treatment. The experiment was repeated three times.

Compared to the control group, both AKAP95 and Cx43 were decreased by H89 treatment (Fig. 7, A and B; *p* < 0.05) and significantly increased by Forskolin (Fig. 7, A and B; *p* < 0.05). Both proteins were still found to interact following treatment with either drug. However, their binding capacity was reduced by H89 treatment and increased by Forskolin treatment (Fig. 7C).

To determine if protein binding capacity was related to protein expression level or PKA phosphorylation, we treated A549 cells with alkaline phosphatase (ALP). The binding capacity of AKAP95 and Cx43 was remarkably increased by ALP treatment (Fig. 7D).

Potential effects of PKA on AKAP95 and Cx43 co-localization was determined by immunofluorescence in A549 cells treated with H89 or Forskolin for 24 h. Samples were examined by confocal laser scanning microscopy, and total counts of H89- and Forskolin-treated cells were respectively 578 and 554, including 517 and 498 in G1/s phase, 19 and 30 in G2 phase, 14 and 8 in prophase, 16 and 15 in metaphase, 7 and 8 in anaphase, and 6 and 5 in telophase.

**Figure 8:**
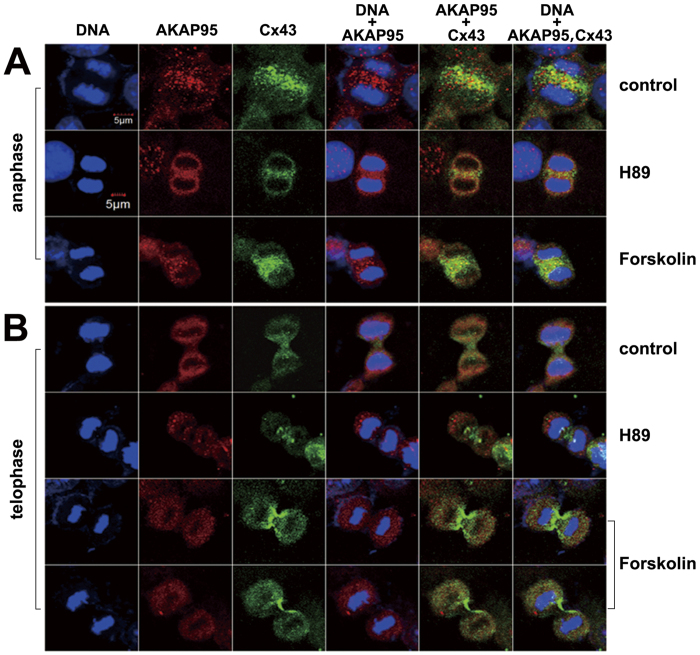
Binding and localization of AKAP95 and Cx43 in A549 cells at different cell cycle phases after H89 and Forskolin treatments. (200 × 3) 1. In anaphase, binding between AKAP95 and Cx43 decreased; only a small portion of these proteins were bound to one other in the H89 and Forskolin treatment groups compared to the control group (**A**); in telophase, there was no binding between AKAP95 and Cx43 (**B**). 2. In anaphase, aggregation of Cx43 between two daughter nuclei was weakened by H89 treatment and enhanced by Forskolin treatment (**A**). 3. After Forskolin treatment, Cx43 was clearly distributed on the cell membrane from the center of the cleavage furrow along the two daughter cells (**B**). This Cx43 localization might be enhanced by Forskolin treatment. The experiment was repeated four times.

In the H89 treatment group, expression of both proteins (especially AKAP95) was decreased. However, no significant changes in protein expression were observed upon Forskolin treatment. Furthermore, there were no changes in protein co-localization with either drug. It should be noted that in the Foskolin treatment group, an increased amount of Cx43 assembled between two daughter nuclei in anaphase (Fig. 8A); moreover, Cx43 was distributed from the center of the cleavage furrow to the cell membrane of the two daughter cells in telophase (Fig. 8B). These results indicated that Cx43 accumulated between two daughter cell nuclei during anaphase. During telophase, Cx43 then gradually moved to the cell membrane of the division sulcus. Forskolin may promote Cx43 function during this transfer process.

## Discussion

Cx43 has multiple biological functions, including regulation of cell proliferation and differentiation, and mainly acts via gap junction intercellular communication and signal transduction^20^,^21^,^22^,^23^,^24^,^25^,^26^. AKAP95 is primarily involved in DNA concentration and gene expression during mitosis^1^,^2^,^3^,^5^,^6^,^7^,^8^,^9^,^10^,^11^. Both Cx43 and AKAP95 affect cell cycle progression by regulating cyclin-CDK complexes^4^,^12^,^26^. Cx43 is a tumor suppressor, and AKAP95 functions to promote tumor growth^27^. Whether Cx43 and AKAP95 directly interact to co-regulate cyclin-CDK activity and affect cell cycle progression is currently unknown. Our results demonstrate that Cx43 and AKAP95 interact in A549 cells in a dynamic fashion that changes during cell cycle progression.

### Cx43 lacks a nuclear localization sequence

However, it has been found in the nucleus of several tumor tissue samples^40^. We used confocal microscopy to detect AKAP95 and Cx43 protein co-localization in the lung cancer cell line A549 and observed dynamic interaction between the two proteins that fluctuated with the cell cycle. Cx43 and AKAP95 expression were both low in early and mid-G1 phase. Following this, expression of the two proteins began to increase (AKAP95 increased slightly earlier than Cx43). AKAP95 was located in the nucleus and Cx43 was mainly found in the cytoplasm. At late G1, AKAP95 and Cx43 were found to co-localize at the nuclear membrane. Cx43 then translocated to the nucleus, where it co-localized with AKAP95 and DNA. This complex was maintained until the end of G2 phase and the start of M phase. During this time, AKAP95 participated in formation and concentration of chromosomal DNA and helped maintain DNA concentration^1^,^11^,^41^,^42^. Additionally, interaction between Cx43 and AKAP95 provided strong evidence that Cx43 also played an important role in DNA concentration and regulation of gene expression. Cx43 and AKAP95 were separated from condensed chromosome during M phase. During this time, the nuclear membrane was broken down and the equatorial plate formed. AKAP95 was still found to play a key role in maintenance of DNA concentration and gene regulation^28^,^29^,^30^; thus, Cx43 may also play a key role in this process. Expression of both Cx43 and AKAP95 began to decrease at anaphase, and Cx43 was concentrated between two daughter cell nuclei. During telophase, Cx43 gradually accumulated at the cell membrane of the division sulcus. This finding is consistent with a previous report from Boassa *et al.*^43^. Dynamic changes in Cx43 revealed that this protein participates in the division of nuclei during anaphase and in the formation of the cell membrane during telophase; neither of these processes required AKAP95. After telophase, both proteins show decreased expression until the next G1 is reached and the cycle begins again. Importantly, co-localization of AKAP95 and Cx43 at the nuclear membrane appears to form as “plaques”; this was supported by results from the proximity ligation assay. Similar findings for Cx43 at the cell membrane have also been reported^44^. Whether or not Cx43 can form a dimer or 6-mer following loading into the nucleus requires further study.

### AKAP95 is an anchor protein for PKA

Thus, we examined whether PKA phosphorylation plays role in Cx43 binding to AKAP95. We used Forskolin and H89 to increase and inhibit PKA activity, respectively. We found that while Forskolin increased binding between the two, H89 reduced their interaction. There are two possible reasons for these observations: 1) phosphorylation of PKA could promote their interaction; 2) Forskolin increases expression of the two proteins while H89 reduces their expression. Treatment with ALP enhanced interaction between AKAP95 and Cx43, which supports the second hypothesis that phosphorylation is not required. This seems to be consistent with an earlier report from Philippe Collas^1^. During interphase in HeLa cells, AKAP95 does not bind to the RII subunit of PKA. However, the two interact in mitosis. PKA is a cAMP-dependent protein. Forskolin enhances PKA activity by increasing cAMP levels^45^,^46^, and H89 reduces the local concentration of cAMP by inhibiting PKA activity^47^.Therefore, the increased expression of AKAP95 following Forskolin treatment may be due to an increase in cAMP levels. An increase in AKAP95 expression results in increased Cx43 binding. Inhibition of PKA triggers a feedback response to reduce cAMP levels, which may subsequently decrease the interaction. It is possible that dynamic changes in interaction between AKAP95 and Cx43 during the cell cycle are regulated by fluctuations in levels of cAMP. Additionally, phosphorylation may serve as a switch for the interaction between AKAP95 and Cx43. Regulation of interaction between AKAP95 and Cx43 at different stages of the cell cycle and its biological significance may be more complex than originally thought; as such, additional studies are required.

Collas *et al.*^1^ reported that a small amount of AKAP95 existed on DNA during M phase. We find that AKAP95 is primarily located around concentrated DNA during M phase, and that a small amount of AKAP95 can also be observed on DNA. Although our findings correlate with those of Collas, we consider that this small amount of AKAP95 on DNA was left over from the process of AKAP95 separating from DNA and then transferring to surrounding DNA.

An improved understanding of the dynamic changes in the expression and interactions of AKAP95 and Cx43 during the cell cycle could provide the foundation for further studies of the function of these two proteins. Our previous studies have shown that AKAP95 protein expression is higher in human lung cancer and esophageal cancer tissues than that in pericarcinoma tissues^48^,^49^, indicating that the AKAP95 protein may play a role in promoting cancer. Cx43 protein is also known to inhibit cancer^15^,^17^,^20^,^21^,^23^,^25^,^26^,^27^. Furthermore, AKAP95 and Cx43 are involved in regulation of the cell cycle by affecting cyclin-Cdk complexes. Therefore, there is no doubt that the interaction between AKAP95 and Cx43 plays an important role in the regulatory mechanism responsible for “promoting and inhibiting” cell cycle progression. In normal cells, the interaction between AKAP95 and Cx43 is likely to be important in regulating cell cycle progression and that disruption of the interaction between the two proteins will promote the cell cycle, resulting in carcinoma. The specific mechanisms underlying the interactions and functions of AKAP95 and Cx43 require further investigation.

## Additional Information

**How to cite this article**: Chen, X.X. *et al.* Dynamic changes in protein interaction between AKAP95 and Cx43 during cell cycle progression of A549 cells. *Sci. Rep.* **6**, 21224; doi: 10.1038/srep21224 (2016).
